# “Prevention Is Better Than Cure”: A Plea to Emphasize the Learning Function of Competence Committees in Programmatic Assessment

**DOI:** 10.3389/fvets.2021.638455

**Published:** 2021-04-29

**Authors:** Harold G. J. Bok, Cees P. M. van der Vleuten, Lubberta H. de Jong

**Affiliations:** ^1^Department of Population Health Sciences, Faculty of Veterinary Medicine, Utrecht University, Utrecht, Netherlands; ^2^Department of Educational Development and Research, Maastricht University, Maastricht, Netherlands

**Keywords:** mentoring, remediation, competence committees, programmatic assessment, competency-based education, high-stakes decision-making

## Introduction

Competency-based education (CBE), with an emphasis on learner-centeredness and recurring assessment to monitor students' performance, is widespread in health profession education (HPE) (1–5). CBE is based upon the proposition that an individual learner is able to follow his own unique learning trajectory for each aspect or competence domain. It aims to facilitate context-dependent performance development from novice to competent based on the learner's needs, unique talents, and ambitions. Assessment of clinical competence is a key issue in CBE (6). CBE in the health profession moves beyond the knowledge domain and typically involves multiple cognitive, psychomotor, and attitudinal/relational skills, and attributes. This requires a systematic approach to assessment as single-assessment methods cannot capture all these dimensions (7). Programmatic assessment (PA), as introduced by van der Vleuten et al. (8) in 2012, was recently described as one of five relevant components for evaluating the implementation of competency-based programs (9, 10) and was increasingly implemented worldwide (11–13). PA provides a framework founded on a set of empirical principles aimed at fostering learning in conjunction with robust high-stakes decision making (14–16). Recently, a consensus was reached on 12 theoretical principles of PA (15, 16) These principles can be grouped into three overarching themes (see Box 1) (16).

Box 1 **Theoretical principles of PA grouped into three overarching themes.**

- ***Continuous and meaningful feedback to promote a dialogue with the learner*** ***for the purpose of growth and development****:* (1) Every (part of an) assessment is but a data point (an assessment data point refers to all activities, e.g., tests/presentations/essays, that provide the learner with relevant information about performance; (2) Every data point is optimized for learning by giving performance-relevant information, that is, meaningful feedback, to the learner; (3) Intermediate review is conducted with the purpose of informing the learner on their progression; (4) The learner has recurrent learning meetings with (faculty) mentors informed by a self-analysis of all assessment data; and (5) Programmatic assessment seeks to gradually increase the learner's agency and accountability for their own learning by being tailored to individual learning priorities.
- ***Use of a mix of assessment methods across and within the context of a*** ***continuum of stakes****:* (6) Pass/fail decisions are not made based on a single data point; (7) There is a mix of methods of assessment; (8) The choice of a given method depends on the educational justification for using that method; (9) The distinction between summative and formative is replaced by a continuum of stakes.
- ***Equitable and credible decision-making processes including principles of*** ***proportionality and triangulation****:* (10) Decision-making on learner progress is proportionally related to the stake; (11) Assessment information is triangulated across data points toward an appropriate framework; (12) High-stakes decisions, e.g., promotion and graduation, are made in a credible and transparent manner, using a holistic approach.

For most clinical programs with PA, high-stakes decisions about promotion or licensure are executed by a competence committee based on the review of aggregated assessment data collected over time (16). Different implementations of competence committees are seen across HPE (16, 17) Increasingly, these committees in CBE are a source of debate in the literature. They feature various guidelines regarding the expertise of those involved in making high-stakes judgments (18–20), the decision-making processes (18, 20, 21), and the quality of the information on which the judgments are based (18, 20). All are relevant guidelines and related to the “assessment of learning” function of competence committees. In this paper, however, we would like to make a plea for an increased emphasis on the potential learning function and formative involvement of competence committees during students' training.

## Value Of Performance-Related Information

Information derived from recurrent performance in a learning environment can support education in different ways. It allows for monitoring, guiding, and evaluating multiple affective, behavioral, and cognitive performance dimensions (e.g., roles, competency domains, activities, and learning outcomes).A programmatic approach to assessment can yield a variety of performance-relevant information (PRI) that allows progress to be monitored (22). PRI serves as feedback and can be expressed as both qualitative (e.g., narrative feedback documented in a mini-Clinical Evaluation Exercise [mini-CEX]) and quantitative (e.g., objective structured clinical examination scores [OSCEs]) data that inform students about their performance within a certain context, aiming to serve as input for self-directed and deliberate learning (22). A rich repository of PRI collected over time will provide students with a wealth of input that will help them plan activities that foster development toward graduate outcome abilities and performance of safe patient care (23).

Insight into students' performance and development toward program outcomes serves as a foundation for mentoring strategies (24). Mentoring offers guidance in interpreting PRI and supports the co-creation and scaffolding of learning processes (25). PRI then serves as input for evaluation processes, that is, high-stakes decision making. A context-rich, mixed-method repository of PRI from different perspectives potentially increases the breadth and quality of feedback and consequently improves the trustworthiness of high-stakes expert judgment. The utilization of PRI as informative feedback creates the opportunity for just-in-time monitoring, guidance, and remediation of the students' progression. Furthermore, to support students in their educational journey, predictive models derived from students' PRI and historical PRI from their peers could provide additional valuable input (e.g., through the application of artificial intelligence [AI] techniques in relation to narrative text mining and quantitative data analyses) (26). This means that in addition to criterion-referencing applications, that is, comparing performance against predefined criteria, norm-referencing applications of assessment have the potential to formatively inform learners about their potential future performance and simultaneously provide tailored guidance for further individual improvement. PRI allows the students and their mentor to readily identify those areas where they are falling behind. When scaffolding strategies initiated between the student and mentor do not have the anticipated effect, a supportive competence committee that provides just-in-time and improvement-focused guidance could play an important role.

## Context That Shaped Our Perception Of High-Stakes Decision Making

In 2010, a programmatic approach to assessment was implemented in a 3-year competency-based veterinary curriculum in the Netherlands that was mainly organized around clinical rotations (11, 23). Over the last decade, several scientific reports have appeared that describe in detail the different elements of the assessment program (4, 23, 27). In short, the program sought to motivate students to collect PRI through workplace-based assessments (WBAs). These WBAs were used to document supervisors' direct observations and feedback. Both qualitative and narrative types of feedback as well as progression identifiers based on predefined milestones, that is, a rubric with criteria defined on a five-level progression scale, were included as PRI. All PRI was stored in a digital repository for which the students themselves were responsible. The repository and WBAs were constructively aligned around competency domains as described in the VetPro competency framework (28). During their clinical training, students had regular individual meetings (twice a year) with a mentor. These meetings were used to discuss progress and formulate new learning goals based on the learners' reflective analyses of PRI.

After 2 years and at the end of the program, two members of a competence committee independently performed a high-stakes judgment for promotion and/or licensure purposes based on their judgment of PRI across outcomes and rotations over time (27). The judgment resulted in narrative comments on each learning outcome (e.g., competency domain), reports on the strengths, points for improvement, and a grade on a 4–10 scale, with 6 or higher indicating that the student had passed. Research indicated that the rate of consensus between the independent portfolio assessors was substantial, students collected a sufficient amount of PRI, and saturation of information within the repository was attained (27). On average, a high-stakes judgment of a given student's repository took about 45 min. If the assessors disagreed, a third independent assessor was called upon. With at least two assessors judging each repository and with a cohort of approximately 200 students, the process of high-stakes decision making was time intensive. A limited number of students failed by scoring a grade below 6. These students were obliged to remediate on those areas where they fell short by formulating an individual remediation plan in collaboration with their mentors. The experiences gained over the last decade as a member of the competence committee (HB) and researching the effectiveness of this high-stakes decision making process (LJ, HB, and CV) made us realize that postponing the remediation process until the end of a certain trajectory was ineffective, both from the student perspective and from the organization perspective. Therefore, a significant change in how we think about and operationalize competence committees is required.

## Just-In-Time And Improvement-Focused Responsibility For Competence Committees

One of the foundational elements of PA is that it seeks to gradually increase the learners' agency and accountability for their own learning by being tailored to individual learning priorities (principle 5) (15). This self-directed process is then facilitated by a mentor or coach and guides the learners to evaluate their feedback and consider their development over time in terms of the program outcomes (principle 4: the learners have recurrent learning meetings with [faculty] mentors informed by self-analysis of all assessment data) (15, 24, 25). If a learner falls short of certain outcomes, based on predefined outcome threshold data and historical cohort data, scaffolding strategies could be identified, and just-in-time remediation could be provided by the mentor. However, when the intervention undertaken by the student and mentor does not result in the anticipated effect, more assistance is required. We argue that this could be organized through the formation of a competence committee that is optimized to provide just-in-time remediation.

**Figure 1:**
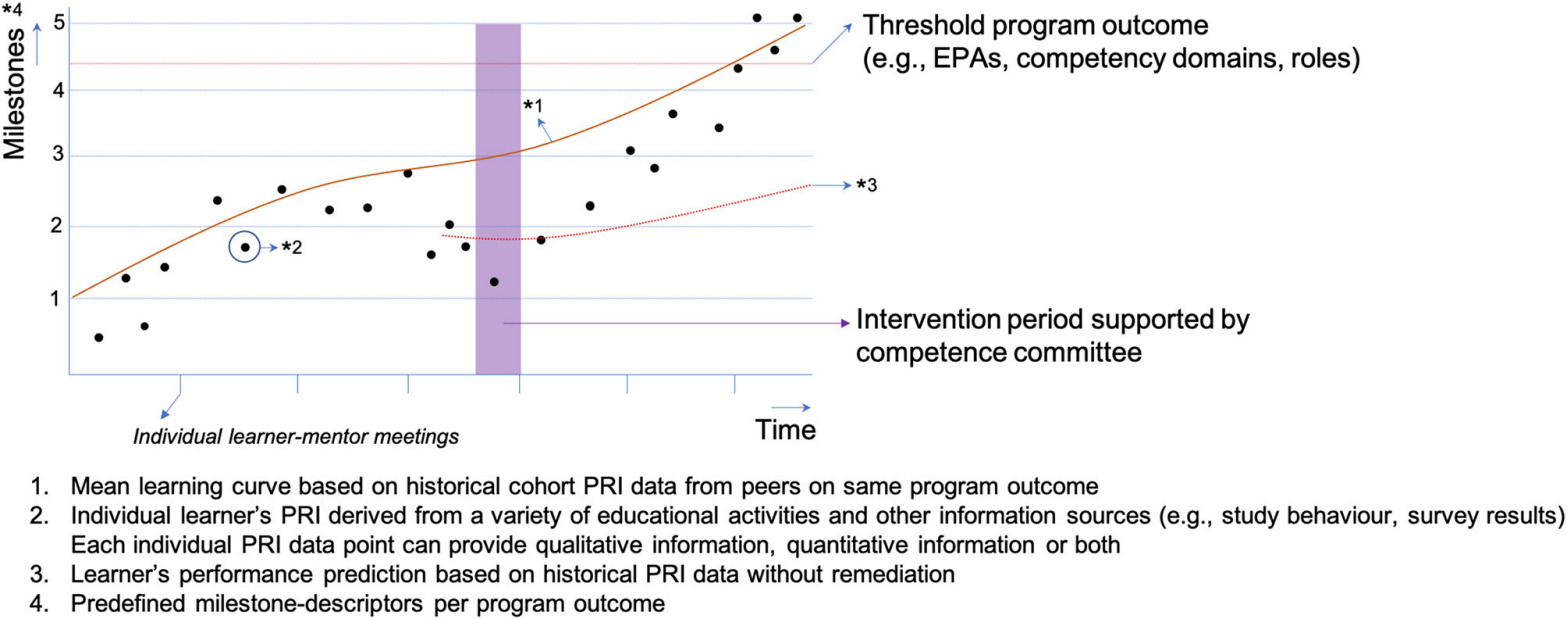
The proposed just-in-time and improvement-focused approach of competence committees in programmatic assessment.

Competence committees set up for just-in-time remediation can potentially have different constitutions. A committee could consist of the learner, the learner's mentor, an experienced independent staff member, and staff from the student support office, as these people can identify the learner's personal needs for remediation in a timely manner. As CBE emphasizes learner-centeredness and is based on the notion that individual learners can follow their own learning trajectory, it is crucial that the students themselves are a member of the competence committee. This will create a shared commitment to fulfilling the remediation. Depending on the outcome at stake, just-in-time remediation can have a variety of forms, such as, retaking an exam, redoing (parts of) a clerkship, writing an essay, attending a communication course, attending a resilience workshop, or attending a learn-to-learn workshop. As this is unexplored territory, future research should focus on how to create conditions that allow these committees to succeed, such as how to build a shared mental model between committee members and create a culture that promotes constructive committee discussions about how to optimally remediate performance. Figure 1 provides a schematic overview of how this could work in practice.

High-stakes decision making in HPE is often operationalized by forming groups of experts who judge at the end of training repositories that contain a multitude of assessment data points (16). As a consequence, in most implementations, the “assessment of learning” function is at the heart of the activities executed by competence committees. However, if we agree that successfully fulfilling the educational journey will guarantee that the outcomes are met at the end of the training, this could have important consequences for how competence committees are operationalized in CBE. The shift in focus toward guidance and just-in-time remediation of competence committees could help steer efforts and investments to assist learners in attaining program outcomes. When successfully implemented, the formal, high-stakes decisions about promotion, and/or licensure at the end of the training could theoretically be replaced by a procedural check.

However, there remain some important questions that require further research. For example, more insight is needed on how just-in-time remediation should be organized. How can mentoring be optimized to prevent the deployment of a competence committee, who are the members of these so-called supportive committees, how can a culture of co-creation and improvement be created, what are the most effective procedures for optimizing the utility of these committees, when should a committee be discharged, and what measures can be taken to limit the tensions that occur while combining the decision making and learning function of assessment? As shown in a recent review by Schut et al. (29) tensions will emerge when simultaneously stimulating the development of competencies and assessing results. It is likely that students will consider the just-in-time intervention by the committee to be summative. This issue was also addressed by Sawatsky et al. (30) who acknowledges the importance of coaching models in clinical education, with its focus on an orientation toward growth and embracing failure as an opportunity for learning, as a potential solution. Therefore, future research and evaluations of innovative implementations need to provide new insights on how to operationalize this just-in-rime remediation. Finally, more research is needed on the robustness of the high-stakes judgment at the end of the training. Is it really sufficient to only have a procedural check at the end of the training, who are involved in that process, what quality measures need to be incorporated to assure all learners have met the program outcomes at the end of the training, and how do learners perceive this method of high-stakes decision making?

## Discussion

In this paper, we aimed to present a different and innovative approach based on our own experiences on how to more meaningfully operationalize competence committees within HPE. Emphasizing the “assessment for learning” role of competence committees could have multiple positive outcomes. It could support learners in taking the lead in their own learning. It could also steer assessment activities toward fostering learning by providing relevant feedback, which could help reduce the tension between formative and summative assessment applications. PRI (i.e., both qualitative and quantitative types of feedback) could potentially be utilized to create a path toward remediation. The application of competence committees in designing just-in-time remediation could, ultimately, foster the development of an educational culture that is focused on the improvement of performance rather than on the assessment of performance (31).

## Author Contributions

HB drafted the initial version of the manuscript and the other authors were involved with editing the manuscript and approved the final version. All authors made substantial contributions to the design of the study and agree to be accountable for all aspects of the work.

## Conflict of Interest

The authors declare that the research was conducted in the absence of any commercial or financial relationships that could be construed as a potential conflict of interest.

## References

[1] Carraccio C Wolfsthal SD Englander R Ferentz K Martin C. Shifting paradigms: from Flexner to competencies. Acad Med. (2002) 77:361–7. 10.1097/00001888-200205000-00003 12010689

[2] Frank JR Snell LS ten Cate O Holmboe ES Carraccio C Swing SR. Competency-based medical education: theory to practice. Med Teach. (2010) 32:638–45. 10.3109/0142159X.2010.501190 20662574

[3] Frenk J Chen L Bhutta ZA Cohen J Crisp N Evans T. Health professionals for a new century: transforming education to strengthen health systems in an interdependent world. Lancet. (2010) 376:1923–58. 10.1016/S0140-6736(10)61854-5 21112623

[4] Bok HG. Competency-based veterinary education. Perspect Med Educ. (2015) 4:86–9. 10.1007/s40037-015-0172-1 25814329 PMC4404455

[5] Matthew SM Bok HG Chaney KP Read EK Hodgson JL Rush BR. Collaborative development of a shared framework for competency-based veterinary education. J Vet Med Educ. (2020) 47:578–93. 10.3138/jvme.2019-0082 32530802

[6] Iobst WF Sherbino J ten Cate O Richardson DL Dath D Swing SR. Competency-based medical education in postgraduate medical education. Med Teach. (2010) 32:651–6. 10.3109/0142159X.2010.500709 20662576

[7] Norcini J Brownell Anderson M Bollela V Burch V João Costa M Duvivier R. 2018 Consensus framework for good assessment. Med Teach. (2018) 40:1102–9. 10.1080/0142159X.2018.1500016 30299187

[8] Van der Vleuten CP Schuwirth LW Driessen EW Dijkstra J Tigelaar D Baartman LK. A model for programmatic assessment fit for purpose. Med Teach. (2012) 34:205–14. 10.3109/0142159X.2012.652239 22364452

[9] Van Melle E Jason FR Holmboe ES Dagnone D Stockley D. A core components framework for evaluating implementation of competency-based medical education programs. Acad Med. (2019) 94:1002–9. 10.1097/ACM.0000000000002743 30973365

[10] Iobst WF Holmboe ES. Programmatic assessment: the secret sauce of effective CBME implementation. J Grad Med Educ. (2020) 12:518–21. 10.4300/JGME-D-20-00702.1 32879699 PMC7450746

[11] Bok HG Teunissen PW Favier RP Rietbroek NJ Theyse LF Brommer H. Programmatic assessment of competency-based workplace learning: when theory meets practice. BMC Med Educ. (2013) 13:123. 10.1186/1472-6920-13-123 24020944 PMC3851012

[12] Heeneman S Oudkerk Pool A Schuwirth LW van der Vleuten CP Driessen EW. The impact of programmatic assessment on student learning: theory versus practice. Med Educ. (2015) 49:487–98. 10.1111/medu.12645 25924124

[13] Perry M Linn A Munzer BW Hopson L Amlong A Cole M. Programmatic assessment in emergency medicine: implementation of best practices. J Grad Med Educ. (2018) 10:84–90. 10.4300/JGME-D-17-00094.1 29467979 PMC5821020

[14] Van der Vleuten CP Schuwirth LW. Assessing professional competence: from methods to programmes. Med Educ. (2005) 39:309–17. 10.1111/j.1365-2929.2005.02094.x 15733167

[15] Heeneman S De Jong L Dawson L Wilkinson T. Consensus statement for programmatic assessment – agreement on the principles. Med Teach. (in press) 10.1080/0142159X.2021.1957088 34344274

[16] Torre D Rice N Ryan A Bok HG Dawson L Bierer B. Consensus statement for programmatic assessment – implementation of programmatic assessment consensus principles. Med Teach. (in press) 10.1080/0142159X.2021.1957088 34344274

[17] Duitsman ME Fluit CR van Alfen-van der Velden JA de Visser M ten Kate-Booij M Dolmans DH. Design and evaluation of a clinical competency committee. Perspect Med Educ. (2019) 8:1–8. 10.1007/s40037-018-0490-1 30656533 PMC6382624

[18] Cook DA Kuper A Hatala R Ginsburg S. When assessment data are words: validity evidence for qualitative educational assessments. Acad Med. (2016) 91:1359–69. 10.1097/ACM.0000000000001175 27049538

[19] Wilkinson TJ Tweed MJ. Deconstructing programmatic assessment. Adv Med Educ Pract. (2018) 9:191–7. 10.2147/AMEP.S144449 29606896 PMC5868629

[20] Tweed MJ Wilkinson TJ. Student progress decision-making in programmatic assessment: can we extrapolate from clinical decision-making and jury decision-making? BMC Med Educ. (2019) 19:176. 10.1186/s12909-019-1583-1 31146714 PMC6543577

[21] Driessen EW Heeneman S van der Vleuten CP. Portfolio assessment. In: Dent JA, Harden RMM, editors. A practical guide for medical teachers. Edinburgh: Elsevier Health Sciences (2013). p. 314–23.

[22] Van der Leeuw RM Teunissen PW van der Vleuten CP. Broadening the scope of feedback to promote its relevance to workplace learning. Acad Med. (2018) 93:556–9. 10.1097/ACM.0000000000001962 29068817

[23] Bok HG de Jong LH O'Neill T Maxey C Hecker KG. Validity evidence for programmatic assessment in competency-based education. Perspect Med Educ. (2018) 7:362–72. 10.1007/s40037-018-0481-2 30430439 PMC6283777

[24] Meeuwissen SN Stalmeijer RE Govaerts M. Multiple-role mentoring: mentors' conceptualisations, enactments and role conflicts. Med Educ. (2019) 53:605–15. 10.1111/medu.13811 30723949 PMC6590242

[25] Agricola BT van der Schaaf MF Prins FJ van Tartwijk J. Shifting patterns in co-regulation, feedback perception, and motivation during research supervision meetings. Scand J Educ Res. (2020) 64:1031–50. 10.1080/00313831.2019.1640283

[26] Tolsgaard MG Boscardin CK Park YS Cuddy MM Sebok-Syer SS. The role of data science and machine learning in health professions education: practical applications, theoretical contributions, and epistemic beliefs. Adv Health Scienc Educ. (2020) 25:1057–86. 10.1007/s10459-020-10009-8 33141345

[27] De Jong LH Bok HG Kremer WD van der Vleuten CP. Programmatic assessment: can we provide evidence for saturation of information? Med Teach. (2019) 41:678–82. 10.1080/0142159X.2018.1555369 30707848

[28] Bok HG Jaarsma DA Teunissen PW Van der Vleuten CP Van Beukelen P. Development and validation of a competency framework for veterinarians. J Vet Med Educ. (2011) 38:262–9. 10.3138/jvme.38.3.262 22023978

[29] Schut S Lauren AM Maggio LA Heeneman S Van Tartwijk J Van der Vleuten CPM. Where the rubber meets the road: An integrative review of programmatic assessment in health care professions education. Perspect Med Educ. (2020) 10:6–13. 10.1007/s40037-020-00625-w 33085060 PMC7809087

[30] Sawatsky AP Huffman BM Hafferty FW. Coaching versus competency to facilitate professional identity formation. Acad Med. (2020) 95:1511–4. 10.1097/ACM.0000000000003144 31895702

[31] Watling CJ Ginsburg S. Assessment, feedback, and the alchemy of learning. Med Educ. (2019) 53:76–85. 10.1111/medu.13645 30073692

